# Specificity of Individual Response Radial Increment of Scots Pine in the Voronezh Biosphere Reserve on the Differentiated Forest Conditions

**DOI:** 10.3390/life12111863

**Published:** 2022-11-12

**Authors:** Sergey Matveev, Daria Litovchenko, Alexander Gusev, Yuriy Golovin

**Affiliations:** 1 Faculty of Forestry, Voronezh State University of Forestry and Technologies Named after G.F. Morozov, 8 Timiryazev Street, 394087 Voronezh, Russia; 2 Institute “Nanotechnology and Nanomaterials”, G.R. Derzhavin Tambov State University, 392000 Tambov, Russia; 3 Department of Functional Nanosystems and High-Temperature Materials, National University of Science and Technology “MISIS”, 119991 Moscow, Russia; 4 Department of Chemical Enzymology, School of Chemistry, Lomonosov Moscow State University, 119991 Moscow, Russia

**Keywords:** radial increment, different response, coefficients of synchronicity, climatic signal, heterogeneous microrelief

## Abstract

The purpose of our study was to assess the individual variability of the response to climatic conditions of the radial increment of *Pinus sylvestris* L. trees aged 100–140 years. The studied pine stand grows in the conditions of a site with a heterogeneous microrelief in the Voronezh Reserve. The calculated coefficients of synchronicity and correlation of radial increment of a sample of individual Scots pine trees (wood cores). It has been established that in the radial increment of pine trees in the Voronezh Reserve, there is a significant diversity in the reflection of the climatic signal, which, as a rule, manifests itself in certain years that are not extreme in terms of climatic conditions. The reasons for the differentiated reaction of trees to climate are the differentiated conditions of the microrelief, and also, probably, the genetic diversity of forest stands. In natural stands there are individual trees showing very low values of synchronicity coefficients (GLK, %) or correlation coefficients (CC, %) with stand average values. Intrapopulation differences in the response of pine forest stands to fluctuations in climatic factors are one of the forms of protective mechanisms for the survival of a species that have developed as a result of evolutionary development. As our study showed, intrapopulation differences are large in stands of natural origin and not subject to anthropogenic impacts.

## 1. Introduction

Many publications have already been devoted to the study of the influence of climatic factors on the radial increment of pine stands in various geographical and landscape conditions in the 21st century [1,2,3,4,5,6], including in the forest-steppe and steppe [7,8,9,10,11,12,13]. At the same time, both the predominant influence of the thermal regime on the growth dynamics [14,15] and the atmospheric humidification regime [11,16,17] have been revealed [18]. It was also noted that in arid mountainous conditions, the combined influence of the thermal regime and atmospheric precipitation creates an unstable climatic response [19,20].

As studies in various geographical regions have shown, dendrochronological series (tree-ring chronologies) of the Scots pine under conditions of forest-steppe, forest-steppe-steppe ecotones, island steppe, and forest-steppe pine forests characterized by an insufficient amount and an uneven distribution of precipitation, are characterized by a low sensitivity coefficient [10,21,22] and a relatively high correlation with spring–summer precipitation [21,23,24].

In areas with the predominance of one climatic factor that limits the growth of woody plants, the influence of local growing conditions (relief, thickness of the soil horizon and soil composition, steepness, and orientation of slopes, etc.) on the variability of radial growth is not significantly manifested [25,26,27].

Studies by Babushkina et al. [13] showed that in the forest-steppe of Southern Siberia, regional warming was slow and did not lead to increased droughts, as it was partially offset by an increase in precipitation in the region, which allowed tree species growing in the region (Scots pine (*Pinus sylvestris* L.), Siberian larch (*Larix sibirica* Ledeb.), Silver birch (*Betula pendula* Roth.) to successfully adapt to the changing conditions of the forest-steppe zone of Southern Siberia.

As shown by a dendrochronological study performed by Caminero et al. [6], the response of the maritime pine (*Pinus pinaster* L.) trees at the southern European border of the Mediterranean (Andalusia) to climatic factors and drought is highly plastic in the most arid study area, especially after the 1980s.

In the forest-steppe of the Russian Plain, over the past 30 years (1990–2020), there has been a significant increase in temperatures of the cold period, an increase in the frequency of recurrence of summer and spring–summer droughts, and an increase in climate instability [11,12,28], which creates significant problems for the adaptation of the Scots pine and Silver birch in this region [29,30].

The dynamics of annual growth of trees is widely used for climate reconstruction and forecasts the impact of climate change on phytocenoses. However, the dependence of these indicators on the characteristics of the individual norm of the reaction of trees to various environmental factors and their interaction has not yet been sufficiently studied [31].

The problem of the possibility of adaptation and acclimatization of woody plants to modern climate changes, associated with their individual characteristics, has acquired significant relevance.

## 2. Materials and Methods

**Figure 1:**
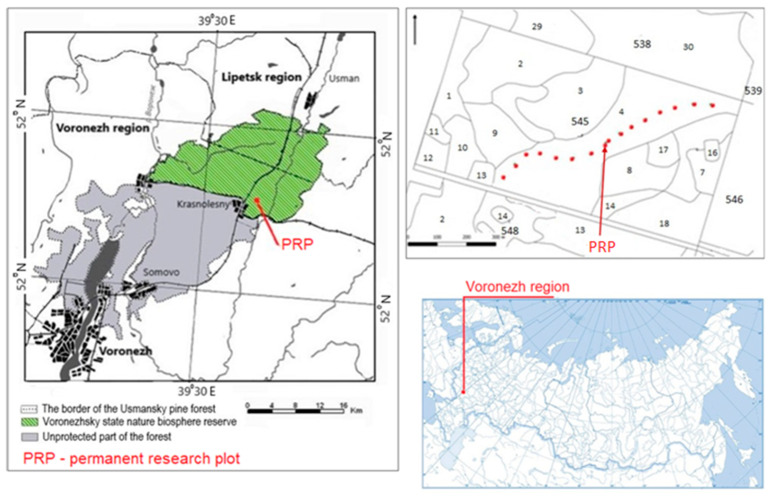
Scheme of the location of the object of study.

The Usmansky pine forest, in which the Voronezh State Natural Biosphere Reserve (Voronezh Reserve) is located, is a natural pine forest with preserved 100–200-year-old stands and minimal anthropogenic impact. Differentiated forest growth conditions are observed here, mainly characteristic of more northern (approximately 100 km) latitudinal conditions. The layout of the objects of study is shown in Figure 1.

In August 2019 and July 2021, we selected 52 wood cores from 26 trees (two cores per tree) in a stand of natural Scots pine, aged 100–140 years old in the Voronezh Reserve, quarter 545, Section 4. It should be noted that the data on the age of trees from cores may contain distortions due to the fact that the drill did not hit the center of the sample, but such cores were excluded in our work. For further analysis, 24 cores were selected from 24 trees that met the requirements for the reliability of the results of dendrochronological studies [32,33,34]. For dating and measuring the width of growth rings, we used the LINTAB-6 measuring complex with the TSAP-Win Professional software package [35].

The forest conditions and microrelief of the section are heterogeneous, not only in the area of the entire section (13 ha) but also within the area of 0.5 ha, where core sampling was carried out. The dendrometric characteristics of the studied stands are given in the Table 1. The distribution of trees by categories of sanitary condition was carried out in accordance with the current scale: 1 (no signs of weakening), 2 (weakened), 3 (very weakened), 4 (shrinking), and 5 (dead) [36]. The degree of the weakening (state) of planting on PRP was determined as the weighted average value of estimates of the distribution of the stock of trees of the different categories of condition.

The value of the weighted average was calculated by the formula:

(1) $$K_{sr}=(P_{1}\times K_{1}+P_{2}\times K_{2}+P_{3}\times K_{3}+P_{4}\times K_{4}+P_{5}\times K_{5})÷100$$

where *K_sr_* is the average value of states categories; *P_i_* is the proportion of trees of each category of condition as a percentage of the stock; and *K_i_* is the index of the tree state category (1—no signs of weakening, 2—weakened, 3—severely weakened, 4—drying out, 5—dead).

The weighted average value of the condition category according to the PRP data is 2.3, i.e., the stand is weakened.

With the help of software TSAP-Win Professional, the coefficients of similarity of the chronologies of the width of annual rings of each sample with the average chronology for the surveyed forest stand were calculated (GLK—synchronism coefficient, GSL—synchronism level); correlation coefficient (CC) between the tree-ring width chronologies of each sample and the average chronology for the stand under study; and cross-dating index (CDI) [32].

We also calculated the coefficient of variation (CV) for the annual ring widths of different trees in the forest stand in different calendar years with different climatic conditions.

The above parameters are calculated as follows.

GLK (S), %—synchronism coefficient, estimates the number of unidirectional changes from year to year between two chronologies [32].

(2) $$S=\frac{n^{+}}{n-1\;}\cdot 100\%$$

where *n*^+^ is the number of annual segments of two chronologies that coincided in direction and n is the duration of the time interval of compared chronologies.

The relatively low synchronicity of changes in the value of radial growth (67% or less) indicates the presence of an intense division of trees within individual dominance ranks. The maximum level of differentiation and variability (79–100%) is observed in years with an optimal combination of environmental factors.

GSL (*) evaluates the level of synchronicity: ≤56 = -; 57–60% = 1 (*); 61–64% = 2 (**); and ≥68% = 3 (***) built individual tree-ring chronologies [32].

Pearson’s correlation coefficient was calculated as a quantitative estimate of the relationship between the studied phenomena (the chronologies of the width of annual rings of each sample and the average chronology for the studied stand).

CC (r_xy_) % is the correlation coefficient: a numerical measure of the strength and direction of a relationship (expressed in %) between two chronologies

(3) $$r_{\mathrm{xy}}=\frac{\sum (x_{i}-\bar{x})\cdot \left(y_{i}-\bar{y}\right)}{\sqrt{\sum (x_{i}}-\bar{\bar{x}{)}^{2}\;\cdot \;\sum (y_{i}-\bar{\bar{y}{)}^{2}}}}\cdot 100\%$$

where

*x_i_*—are the values taken in the sample X;

*y_i_*—are the values accepted in the sample Y;

$\bar{x}$—arithmetic mean for variable X;

$\bar{y}$—arithmetic mean for variable Y.

The relationship is: weak (0–30), moderate (31–50), significant (51–70), high (close) (71–90), very high (very close) (91 or more).

CDI—cross-dating index: an indicator that is a combination of Student’s test (t_st_), correlation coefficient (CC) and synchronicity parameter (GLK), expressed as a percentage (%), calculated in software TSAP-Win Professional [35]. It is customary to recognize CDI values of more than 10% as reliable.

CV—coefficient of variation (relative standard deviation): a standard measure of the dispersion of a probability or frequency distribution. Usually expressed as a percentage and defined as the ratio of the standard deviation (*σ*) to the arithmetic mean (*μ*):

(4) $$\mathrm{CV}=\frac{\sigma }{\mu }\cdot 100\%$$

We calculated the values of the coefficient of variation (CV) for each calendar year, which are the deviations of the width of the annual rings of individual chronologies from the value of the width of the annual ring of the average chronology taken as 100%.

Long-term dendrochronological studies of the Scots pine in the central forest-steppe of the Russian Plain, which we carried out previously [12,24,28], as well as the observed similarity in the reflection of the climatic signal in pine forests growing on fresh and moist sandy loamy soils, allowed us to build generalized chronologies of the second order (master chronologies) for of all large island pine forests of the central forest-steppe – Usmansky, Khrenovskoy, and Tsninsky. We averaged three master chronologies into a summary bar chart dynamics of indices of the radial increment of Scots pine in the central forest-steppe of the Russian Plain on fresh and moist sandy loamy soils depending on climatic factors from 1900 to 2020. 

## 3. Results

**Figure 2:**
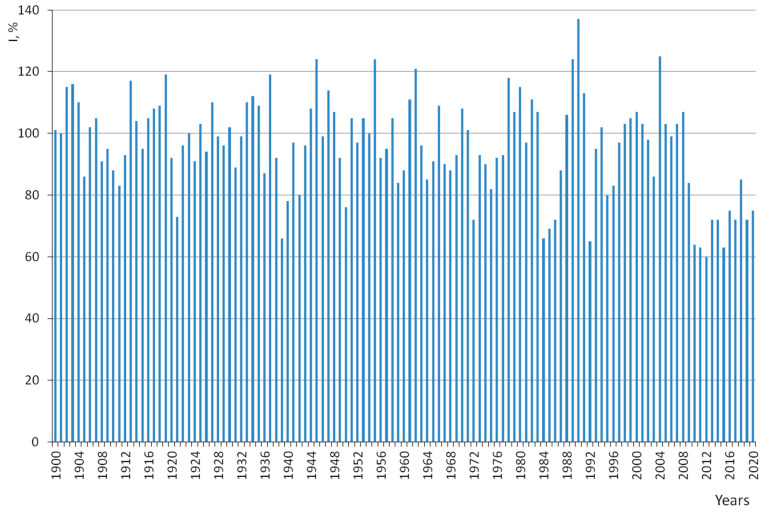
Summary bar chart dynamics of indices of the radial increment of Scots pine in the central forest-steppe of the Russian Plain on fresh and moist sandy loamy soils depending on climatic factors from 1900 to 2020.

The bar chart of the radial increment of the Scots pine in the central forest-steppe is very effective in dating complex cores, wood of unknown origin, and identifying false rings (Figure 2). The bar chart clearly shows the accounting years both with an abnormally large increase (1944–1945, 1980, 1990, 2004) and with an abnormally small increase (1921, 1939, 1972, 1992). However, there are years in which growth is very diverse and can (for different trees) vary from very small to very large (1930, 1943, 1949, 1954, 1969, 1981, 1991, 1995, 2003, 2013), which is reflected by the average values for bar chart.

**Figure 3:**
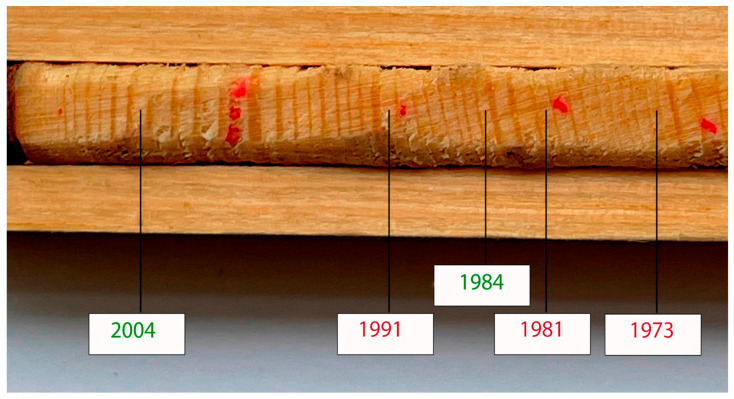
A fragment of a wood core with typical and non-typical annual rings: green color—typical rings; red—atypical. Atypical narrow: 1973; atypical wide—1981, 1991.

An illustrative example of typical and non-typical rings of a wood core fragment for individual years is shown in Figure 3.

During the research, we uncovered features in the reactions of Scots pine stands to climatic factors in heterogeneous forest site conditions (micro- and mesorelief) within the same research plot and in trees growing nearby, in homogeneous forest conditions, obviously related to their genetic diversity. The peculiarities of the reaction of the radial growth of trees to climatic factors, revealed by us in this study, are most noticeably manifested in the conditions of the protected regime of the Voronezh Reserve, which we noted earlier [24].

**Figure 4:**
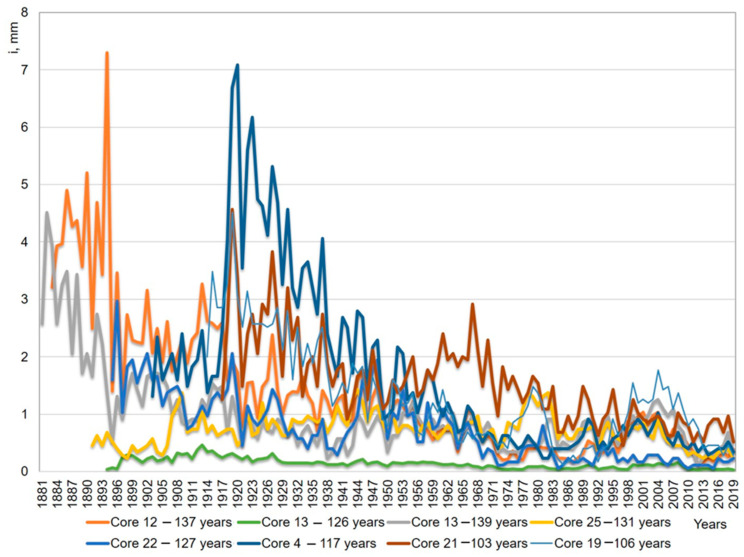
Dynamics of radial increment of trees of different ages in the studied stand.

The comparison of the dynamics of radial increment of trees in the examined forest stand with a summary bar chart for the central forest-steppe, which characterizes the typical responses of radial increment to climatic factors (Figure 2), emphasizes the individual features of the variability of the response of individual trees in the conditions of the Voronezh Reserve (Figure 4).

The age structure of the stand is not parallel to the change in diameters. The two oldest trees (aged 139 and 137 years) on the breast height have the same diameter of 51 cm. The two relatively young of the model trees, aged 103 and 106 years (at a height of 1.0 m), have diameters of 62 and 61 cm, respectively. The smallest diameter, 40 cm, is observed in pine trees aged 126, 127, and 131 years. The largest diameter, 64 cm, is observed in a 117-year-old pine (Figure 4).

**Figure 5:**
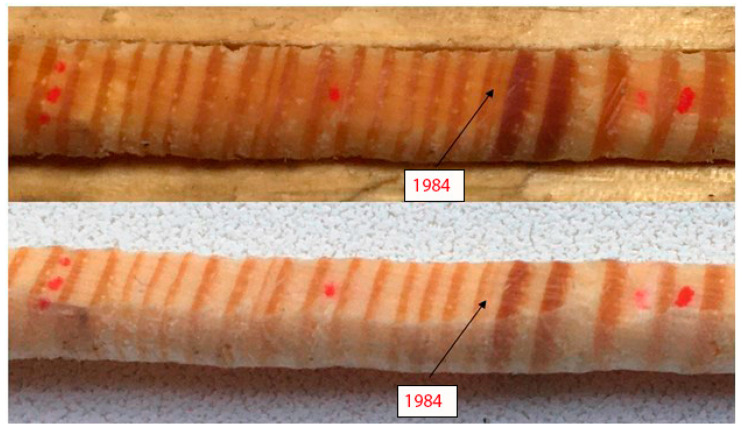
Voronezh Reserve, quarter 545, Section 4, sample No. 25. False ring 1984, dry (top), wet (bottom).

Attention should also be paid to the importance of identifying false and drop-out rings during the dating of wood samples (cores) in Figure 5.

According to Copenheaver et al. false rings may become a new source of indirect data on historical environmental conditions once the reasons for their formation are understood. False rings form predominantly on dominant and co-dominant trees in the stand, with rings formed early in a tree’s life more likely to contain false rings than rings formed later in life [37]. These researchers consider it very important to study the causes of the formation and characteristics of false rings in order to distinguish between false rings formed as a result of the position of the tree in the stand or other individual characteristics and associated with the climatic conditions of the growing season.

The analysis of the values of the synchronicity coefficient (GLK) of individual chronologies of tree rings with an average chronology showed that the range of fluctuations is large: 56–79% (Table 2—the table shows the samples with the lowest and highest values).

One sample, No. 24, showed synchronicity values with the average for both early and total wood (56%) with a missing level of synchronism on the scale of S.G. Shiyatov [32].

Synchronicity average:

in terms of total wood (72%) corresponds to the average level according to the S.G. Shiyatov [32];

for early wood (71%)—the average level;

for late wood (72%)—the average level.

The synchronicity level (GSL) for almost all samples for all types of wood is 3 (***), with the exception of one sample, No. 24, which has no synchronicity for total and early wood (-), and for late wood it has a value of 1 (*).

The correlation coefficient (CC, %) is even more variable than the synchronicity co-efficient. Samples No. 24 and No. 25 for late wood showed values of 21 and 28%, i.e., weak correlation with the mean. For total wood sample, No. 24 showed a value CC = 63% (significant relationship) and for earlywood, CC = 75% (high relationship). Sample No. 25 showed low correlation values for both total and early wood, respectively: CC = 28% (weak relationship) and CC = 31% (moderate relationship).

The average value of the correlation coefficient of total wood: 67% (significant relationship), with a range of fluctuations from 28% (one sample), the rest ranged from 53% to 93%.

The average value of the correlation coefficient of early wood is 70% (significant relationship), with a range of fluctuations from 31% (one sample), the rest ranged from 55% to 91%.

The lowest values of the correlation coefficient are observed for late wood. Average: 60% (significant relationship), with a range of fluctuations from 21 and 28% (two samples), the rest ranged from 34% to 87%.

Sample No. 24 also shows a low value for the cross-dating index (CDI), with earlywood the lowest value at 7%, latewood at 14%, and total wood at a slightly higher value at 18%.

Usually, if the CDI value is less than 10 percent, the sample is excluded from further processing as being erroneously dated. The higher the CDI, the more synchronous response to climatic factors the examined samples have.

However, as our study showed, in real natural forest stands with high genetic diversity, there may be trees with individual responses to climatic factors with CDI values of less than 10 percent.

The average value of the cross-dating index for latewood is the lowest at 47% compared to the total width of the growth ring (57%) and earlywood (52%). The range of fluctuations in the cross-dating index for late wood is significant: from 7% to 84% (Table 2).

**Figure 6:**
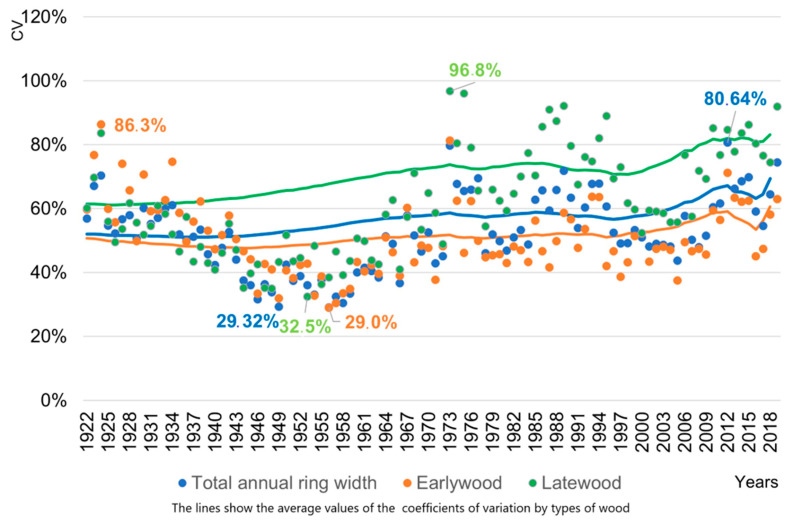
Distribution of coefficients of variation by types of wood between individual chronologies by year.

An analysis of the dynamics of the values of the coefficient of variation by wood types showed that its values vary greatly by calendar year, and the coefficient of variation decreases with age (Figure 6). 

We presented the two highest and lowest values of the coefficient of variation (CV) for each type of wood and the years in which they were observed in Table 3.

The largest values of the coefficient of variation was noted in the two driest years (1924 and 1975) and in the two with the highest level of spring–summer precipitation (1973 and 2012).

The smallest values of the coefficient of variation are mainly observed in years with optimal climatic conditions.

During the lifetime of trees, the coefficient of variation has a range of fluctuations from 29.32% to 80.64% (for total wood). Late wood is the most variable (up to 96.8% of the arithmetic mean). The graph (Figure 6) of the variability of these types of wood clearly shows the increase in the variability of tree ring widths from the 1970s to the present. The increase in the variability in the width of annual rings is due to the increase in the variability of climatic factors in this time period.

Our analysis of the growth dynamics of pine trees in this area showed significant differences in the reflection of the climatic signal, obviously associated with both the heterogeneity of the microrelief and the genetic diversity of the pine stand. 

## 4. Discussion

Ng’andwe et al. [38] and Zhang et al. [39] argue that site-specific ring width chronology and tree growth variation has great potential to study tree responses to edaphic and climatic factors. The knowledge of these reactions deepens our understanding of the growth of pines of various origins under the conditions of climate change.

Intraspecific and intrapopulation differences in the response of pine forest stands to fluctuations in climatic factors are one of the forms of protective mechanisms for the survival of a species that have developed as a result of evolutionary development. Forest stands of natural populations with a rich and diverse gene pool are more resistant to extreme environmental and climate conditions. In the conditions of reserves, in forest stands of natural origin, such diversity is higher and more clearly expressed [24,40].

A multiplicity of researchers, both in Russia and abroad, also came to the conclusion that the individual variability of the response of individual trees in a population to the action of climatic factors may be a consequence of the heterogeneity of forest conditions or competitive relations in the forest stand and, in a population growing in homogeneous conditions, can be considered as a result of genotypic differentiation [41,42].

Rumyantsev et al. [42] propose the consideration of a series of radial tree growth “as a record of the results of a series of experiments carried out by nature, placing a given genotype in different ecological conditions”.

Rygalova’s article [43] discusses information on the variability of the sensitivity coefficient of tree-ring chronologies and traces the heterogeneity of the response of individual trees to the same climatic conditions, which can hardly be explained only by topoecological heterogeneity of precipitation. It is most likely that genetically determined features of the reaction of trees take place here.

A similar situation in terms of the heterogeneity of the microrelief was previously noted by us in one area in the Tsnin forest in the Tambov region, Gorelsky Forestry Enterprise [16]. The heterogeneity of the microrelief manifested itself in the differentiation of ground cover plants: succulents and other herbaceous plants typical of dry conditions grew on tussocks and mounds; and in microdepressions and depressions, mainnik, blueberries, and other plants of wet places grew. The reaction of the radial increment of Scots pine trees of natural origin under these conditions was also differentiated.

After the drought in 2010, we observed the same diversity of reactions in individual trees in artificial pine forest stands subjected to rather intense recreational impact: growth decreased in most trees in 2009 and especially in 2010 and 2011 at all stages of digression. However, at all the stages of digression, one or two trees were found that did not reduce radial increment (moreover, these were different trees in 2009 and 2010) [44].

High intra-population genetic diversity will help ensure the adaptation of trees to climate change, especially at the boundaries of their natural habitats [40]. The forest-steppe of the Russian Plain is the boundary of the distribution of island forests. Scots pine in this region are experiencing a significant impact of climate change (increasing intensity and frequency of droughts, climatic “swings”, etc.).

It Is necessary to use the potential of the high intrapopulation genetic diversity of species (including Scots pine) in reserves, especially those growing in highly differentiated forest conditions (microrelief) to enhance the adaptive capacity of woody plants in a changing climate.

## 5. Conclusions

The main conclusions from the results of our study are given below.

1. In the radial increment of pine trees in the Voronezh Reserve, there is a significant diversity in the reflection of climatic signals;

2. Diversity of the reaction of forest stands is more pronounced in years with favorable climatic conditions; however, it also manifests itself in years with extreme climatic conditions;

3. The reasons for the differentiated reaction of trees to climate, as shown by our studies, are differentiated conditions of the microrelief, and also, probably, the genetic diversity of forest stands;

4. Average values of the correlation coefficient of total wood: 67%, early wood; 70%, late wood; 60%, significant relationship in all cases. Individual samples, No. 2, 24, and 25, show very low correlation coefficients with the average for individual types of wood: 21—31%;

5. Late wood is the most variable: the coefficient of variation up to 96.8% of the arithmetic mean. From the 1970s to the present, there has been greater variability in tree-ring widths due to increasing climate variability.

6. One sample, No. 24, showed synchronicity coefficients values with the average for both early and total wood of 56%, with a missing level of synchronism on the scale of S.G. Shiyatov;

7. Intrapopulation differences in the response of pine forest stands to fluctuations in climatic factors is one of the forms of protective mechanisms for the survival of a species that has developed as a result of evolutionary development. As our study showed, intrapopulation differences are large in stands of natural origin and not subject to anthropogenic impacts.

## Figures and Tables

**Table 1:** The dendrometry characteristics of the studied stands determined by the authors (quarter 545; Section 4), growth class—II.

Object of Study	Composition of Tree Species, %	dbh (cm)	h (m)	f	Standing Volume, m^3^/ha	Age, Years *	K_sr_	FGC **
Data of forest managementfor 2013	100% Scots pine	44	28	0.70	320	125	–	B_2_
Core sampling area	100% Scots pine + European birch	47	29	0.59	347	100–140	2.1	B_2_–B_3_
Average characteristic of tree stand of research plot	100% Scots pine + European birch, English oak, Maple	45	29	0.69	340	130	2.3	B_2_

Note: h—mean height; dbh—mean diameter on breast height; f—density of tree placement in the PRP (corresponds to the canopy density); K_sr_—average value of states categories; *—are given values of age, according to forest management for 2013 and according to dendrochronological analysis in the area PRP No.8; **—FGC, forest growth conditions, B_2_—pine forest on moderately moist sandy loamy soils, B_3_—pine forest on moist sandy loamy soils.

**Table 2:** Values of synchronicity coefficients (GLK, %), correlation coefficients (CC, %), synchronicity level (GSL, *), and cross-dating indices (CDI, %) of the individual chronologies of radial increment of pine trees (total annual ring width, early wood, late wood).

Core Number	GLK, %	GSL (*)	CC, %	CDI, %
Total Annual Ring Width				
1	74	3	93	67
10	71	3	92	60
18	76	3	53	65
23	71	3	63	50
24	56	–	57	18
25	76	3	28	69
26	79	3	80	71
Average values:	**72**	**3**	**67**	**57**
Early wood (EW)				
1	73	3	90	67
4	75	3	89	66
8	78	3	85	84
10	73	3	91	62
18	70	3	55	42
23	67	3	60	42
24	56	–	57	7
25	70	3	31	47
Average values:	**71**	**3**	**70**	**52**
Late wood (LW)				
1	72	3	87	45
2	72	3	21	64
3	74	3	34	58
5	79	3	71	63
9	75	3	85	34
10	66	3	83	41
13	71	3	77	44
18	77	3	38	51
20	67	3	78	43
24	59	1	46	14
25	77	3	28	54
26	73	3	77	51
Average values:	**72**	**3**	**60**	**47**

**Table 3:** The extreme coefficients of variation by the wood types between individual chronologies by year, %.

Wood Type	The Largest Values of the Variation Coefficient		The Smallest Values of the Variation Coefficient	
	Years	Values	Years	Values
Total annual ring	2012	80.64	1949	29.32
Total annual ring	1973	79.72	1946	31.57
Early wood	1924	86.3	1956	29.0
Early wood	1973	81.3	1957	30.4
Late wood	1973	96.8	1953	32.5
Late wood	1975	96.1	1948	34.9

## Data Availability

The data presented in this study are available on request from the corresponding author. The data are not publicly available due to privacy restrictions.
